# Supplementary figures and images for: Regulatory T cells in erythema nodosum leprosum maintain anti-inflammatory function
Source: PLoS Negl Trop Dis. 2022 Jul 22;16(7):e0010641. doi: 10.1371/journal.pntd.0010641 (PMC9348709; doi:10.1371/journal.pntd.0010641)

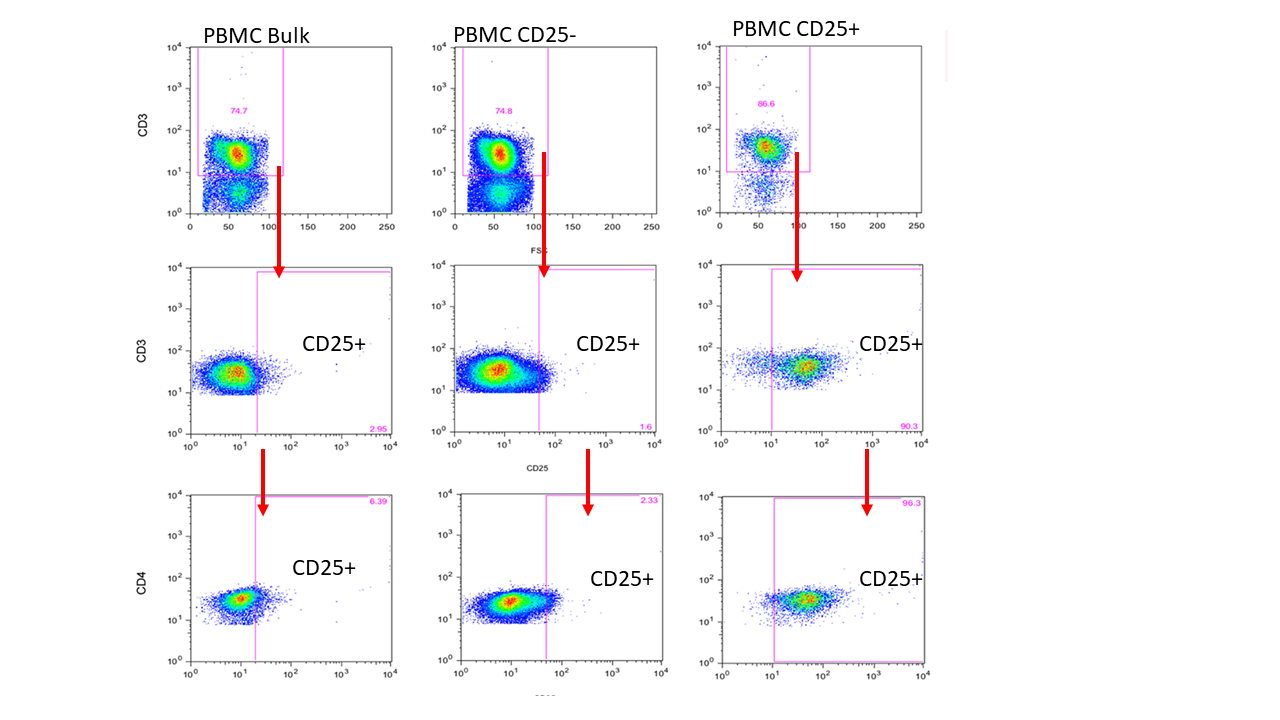

Supplement: S1 Fig — Using magnetic sorter, fractions of each cell population were analysed for their expression of CD 3, CD4 and CD25. (TIF) [file pntd.0010641.s001.tif]
