# Supplementary material for: Regulatory T cells in erythema nodosum leprosum maintain anti-inflammatory function
Source: PLoS Negl Trop Dis. 2022 Jul 22;16(7):e0010641. doi: 10.1371/journal.pntd.0010641 (PMC9348709; doi:10.1371/journal.pntd.0010641)
Supplement: Attachment — Submitted filename: Response to comments PNTD-D-22-00102.docx [file pntd.0010641.s002.docx]

**Manuscript number**: **PNTD-D-22-00102**

**Title**: **Regulatory T cells in Erythema nodosum leprosum maintain anti-inflammatory function**

Response to the reviewers comment

Dear Editor,

We would like to thank the reviewers and the editor for the detailed and useful comments we have received. We have responded to the reviewers’ comments and undertaken a review to improve the manuscript. We have addressed all the comments and hope this is to the satisfaction of the reviewers and editor. We provide below the responses to each reviewer’s comments.

Yours faithfully

Edessa Gobena on behalf of the authors

**Reviewer 1.**

1. **Comment:** The author defined CD25+ PBMC as Treg Cells. However, it is now common to use CD4+CD25Foxp3+ to define Treg Cells. Although there may have been some publications to support, the author should conduct experiment to show the consistence between two methods.

**Response:**

Thank you. We appreciate the concern of the reviewer. However:

*(i). The aim of our study is to investigate if Tregs from LL and ENL are functionally defective or intact. Our experiment was not designed to evaluate the best marker for Tregs.*

*(ii). FOXP3 is an intracellular transcription factor which cannot be sorted out by magnetic sorting unless the cells are permeabilized. Intact and live cells are required for stimulation with M. leprae for this experiment.*

2. **Comment**: Better to add results from skin biopsy.

**Response:** *ENL is a systemic inflammatory disease. Blood samples are ideal for evaluating Tregs for a systemic inflammatory disease like ENL.*

3. **Comment:** There are two leprosy single cell sequencing analysis have been published in Nat Immunology and Cell discovery. Many aspects of leprosy mechanisms especially immunity have been covered. The author should discuss the current results and published results

**Response:** We are aware of the article which was published in January 2022 after our manuscripts has been submitted. “The immune-suppressive landscape in lepromatous leprosy revealed by single-*cell RNA sequencing***”.** <https://doi.org/10.1038/s41421-021-00353-3>. This paper concludes that there is an expansion of Tregs in lepromatous leprosy (LL) patients which means increasing number of Tregs in these patients. Previously we and others have published that the proportion Tregs are increased in LL but significantly reduced in ENL (Attia et al., 2010, Negera et al., 2017). Our current study is evaluating if the reduced proportion of Tregs in ENL is associated with loss of anti-inflammatory function. We cited the article in the introduction section (lines 110-111).

**Reviewer 2**

1. **Comment**: Please add figure of flow cytometry dot plot showing CD25+ and CD25neg depletion assay.

**Response:** *We appreciate the comment and we have added flow cytometry gating to show the percentage of CD25 negative cells achieved after sorting (above 95%) which shows how efficient is our sorting. Suppl Fig 1 line 624-626”* *Flowcytometry data dot plot analysis of bulk (total PBMCs), CD25 depleted and CD25 positive population. Using magnetic sorter, fractions of each cell population were analysed for their expression of CD3, CD4 and CD25*”

2. **Comment:** Treg cells are major source of IL-10 production. This study seems too incomplete without explanation of IL-10 producing cells after the CD25 depletion. Author should add one more flowcytometry experiment to know about IL10 producing cells after 96 hours culture. This may be helpful to further strengthen the current manuscript.

**Response**: *We appreciate the comment. However, the comment is not directly related to the objective of our study for the following reasons:*

*i). The numbers of circulating regulatory T cells (Tregs) are increased in lepromatous leprosy (LL) but reduced in erythema nodosum leprosum (ENL).* *It is unclear whether the suppressive function of Tregs is intact in both these conditions. Therefore, we wanted to investigate if Tregs from LL and ENL are capable of supressing the pro-inflammatory cytokines: TNFα and IFNγ.*

*ii). Tregs supress inflammation in numerous ways (Romano et al., 2019):*

*(a). Through producing anti-inflammatory cytokines (IL-10, IL-35, and TGFβ).*

*(b). By releasing perforin and granzymes, which target cell membrane leading to apoptosis. (c). By depleting IL-2 from the microenvironment through over expression of CD25 (IL-2 receptor) which reduces T cell proliferation.*

*(d). Tregs express CTLA-4 which blocks co-stimulatory molecules (CD80/86) by expression and,*

*(e). By promoting M2 macrophages and preventing the differentiation of pro-inflammatory macrophages, M1.*

*We included a discussion of these mechanisms of T-cell immunoregulation in our discussion section (lines 401-405).*

*As mentioned above, Tregs exert immune suppression in multiple ways other than IL-10. Hence, identifying the source of IL-10 does not provide any further information about the mechanism through which Tregs supress the immune system in lepromatous leprosy or how that suppressive function is impaired in ENL. Our experiment was not designed to study signal transduction of immune cells in ENL or LL and we didn’t do IL-10 staining.* Unfortunately, you do not have cells available to carry out staining for IL-10 in the separated cells populations, but will include this in any further studies.

3. **Comment**: Please also check data analysis test. You should try Wilcoxon T test (Two tailed) for within the groups.

**Response:** *Our data are quantitative continuous data which are normally distributed. We checked for the normality of our data prior to analysis and consulted a statistician. Wilcoxon rank-sum test or Wilcoxon T test (Two tailed) is a non-parametric qualitative test which is less appropriate for our data. When the assumptions (normality and adequate sample size) fulfilled, the t-test which we used has greater statistical power than Wilcoxon test (Wilcoxon T test (Two tailed) ) (Fay and Proschan, 2010). We therefore followed our statistician’s advice and used the t-test.*

4. **Comment**: Graphs need to be more informative like by showing significant differences between the groups. What letter represents what is not mentioned? Before drug (MDT/Prednisolone) and after taking drug, graphs should be shown for more clear information. In the present condition, data presentation is very complicated, it needs to be clear by graphical representation.

**Response:** *In each figure legend the following statements “---Each bar and line graphs show mean ± 95% confidence interval (CI). Different letters indicate statistically significant difference at P ≤0.05 and 95% CI…” lines 235-36; 263-264;285-287; 30-302 and 321-322. To help the reviewer and readers we have further explained the meaning of each letter as: any pair of different letters shows that the mean of the two groups are statistically significantly different at p=0.05 and any pair of similar letters indicates that the mean of the two groups are not statistically significantly different at P=0.05.*

*Using letters in presenting statistical significance in graphs allows multiple pairwise comparisons. For example, 6 pairwise comparison can be made from 4 groups without affecting the readability of the graph. (G1vs G2, G1vs G3, G1 vs G4, G2 vs G3, G2 vs G4 and G3 vs G4). Using letters allows pairwise comparison among several groups and is standard reporting practice used in other peer-reviewed publications including other PLoS journals (for example, Virgilio et al., 2016, Xing et al., 2015, Fathi et al., 2021).*

*For example, in the following figure, taken from our manuscript, up to 36 pairwise comparisons can be made (principles of permutation in statistics: (9x8)/2=36.*

*One can compare PBMC (media) with either PBMC (PHA), PBMC (M. leprae), CD25^-^(media), CD25^-^(PHA), CD25^-^(M. leprae), CD25:2550(media), CD25:2550(PHA) and CD25:2550(M. leprae), altogether 8 pairwise comparisons. Similarly, for PBMC(PHA) 7 pairwise comparisons can be made and for PBMC (M. leprae) 6 pairwise comparisons can be drawn.*

***How to use the letters?*** *For example, PBMC (media) and CD25^-^(media) are both denoted by a letter* ***“a”.*** *this means the mean of IL-10 production in PBMC (media) and CD25^-^(Media) is not statistically significantly different at P=0.05. on the other hand, PBMC (media) is denoted by letter “a” and PBMC (PHA) by letter “b” which is interpreted as the media of IL-10 production in PBMC (Media) and PBMC (PHA) are statistically significantly different at P=0.05.*

5. **Comment:** Authors reported that the function of Treg cells remains intact despite their low number in ENL cases. This needs to be explained in the results and discussion accordingly.

**Response:** *We described in detail that Tregs maintain their immune suppression capability despite reduction in number (line 403-408) “The TNFα response was significantly decreased in autologous CD25^+^ supplemented PBMCs confirming that the suppressive function of CD25^+^ T cells is intact in ENL despite their reduction in number. Similarly, the IFNγ response was significantly enhanced in CD25^+^ depleted PBMCs from untreated individuals with ENL compared to the control PBMCs and the response was reduced by the reintroduction of autologous CD25^+^ cells to CD25^+^ depleted PBMCs” and discussed possible mechanism of immunosuppression by Tregs in ENL (lines 415--424) “In the present study, we report increased levels of pro-inflammatory cytokines (TNFα and IFNγ) in CD25^+^ depleted PBMCs before and after prednisolone treatment and this was abolished by the re-addition of autologous CD25^+^ cells to CD25^+^ depleted PBMCs. This confirms that the relative numerical reduction of Tregs in ENL (Negera et al., 2017) is not associated with a reduced anti-inflammatory function .*

*The ability of Tregs to suppress proinflammatory cytokines in leprosy may be achieved by inhibiting the production of T-cell derived cytokines, by inhibiting monocyte-driven proinflammatory cytokines or through direct apoptosis of activated T cells. Thus, the mechanism through which Tregs downregulate pro-inflammatory cytokines (TNFα and IFNγ) in M. leprae WCS stimulated cells in LL and ENL needs further investigation” (line 415-419).*

6. **Comment:** Regarding IL10 level, line no. 253 - 258 needs to be rewritten as there is no difference in their meaning.

**Response:** Thank you. We have corrected this as “*There was no evidence of a difference in IL-10 responses to M leprae WCS stimulation in CD25^+^ depleted PBMCs or control PBMCs after 24 weeks of MDT compared to pre-treatment in individuals with uncomplicated LL*” in lines 262-266.

**Reviewer 3.**

1. **Comment:** Authors used many outdated references

**Response:** *We respectfully note that we have used appropriate references because of their innovative nature. They are classic references which are key references in this field*

*i). Ridley and Jopling, 1966 (reference 1). We used this article when we described the 5 clinical types of leprosy. Ridley and Jopling classified leprosy in to 5 clinical types called spectrum.*

*ii). Wemambu et al 1969 (reference 6): the clinical manifestation of ENL was compared with the Arthus phenomenon for the first time by these authors and we acknowledged the authors while describing the clinical manifestation of ENL.*

*iii). Mabalay et al, 1965 (reference 7). Mabalay et al are the first and the only authors to describe the sequential development of immune cells which correlate with the duration of ENL lesions.*

*iv). Modlin et al 1983 (reference 12). These authors for the first-time characterized T lymphocyte subsets in situ in ENL skin lesions which laid a foundation for the immunology of T-cells in leprosy reaction.*

2. **Comment:** Table 1: Demographic characteristics of study participants LL(n=30) column Sex row Male=27 and Female=7 the sum of sex=34 not 30

**Reponses**: Thank you. We have corrected this typing error to male 23 (76.6%).

**References**

ATTIA, E. A., ABDALLAH, M., SAAD , A. A., AFIFI , A. & TABBAKH, A. 2010. Circulating CD4+ CD25highFoxP3+ T cells vary in different clinical forms of leprosy. *International Journal of Dermatology* 49**,** 1152-1158.

FATHI, R., AKBARI, A., NASIRI, K. & CHARDAHCHERIK, M. 2021. Ginger (Zingiber officinale roscoe) extract could upregulate the renal expression of NRF2 and TNFα and prevents ethanol-induced toxicity in rat kidney. *Avicenna journal of phytomedicine,* 11**,** 134-145.

FAY, M. P. & PROSCHAN, M. A. 2010. Wilcoxon-Mann-Whitney or t-test? On assumptions for hypothesis tests and multiple interpretations of decision rules. *Statistics surveys,* 4**,** 1-39.

NEGERA, E., WALKER, S. L., BOBOSHA, K., HOWE, R., ASEFFA, A., DOCKRELL, H. M. & LOCKWOOD, D. N. 2017. T-cell regulation in Erythema Nodosum Leprosum. *PLOS Neglected Tropical Diseases,* 11**,** e0006001.

ROMANO, M., FANELLI, G., ALBANY, C. J., GIGANTI, G. & LOMBARDI, G. 2019. Past, Present, and Future of Regulatory T Cell Therapy in Transplantation and Autoimmunity. *Frontiers in Immunology,* 10.

VIRGILIO, S., CUPERTINO, F. B., BERNARDES, N. E., FREITAS, F. Z., TAKEDA, A. A., FONTES, M. R. & BERTOLINI, M. C. 2016. Molecular Components of the Neurospora crassa pH Signaling Pathway and Their Regulation by pH and the PAC-3 Transcription Factor. *PLoS One,* 11**,** e0161659.

XING, J., WANG, G., ZHANG, Q., LIU, X., GU, Z., ZHANG, H., CHEN, Y. Q. & CHEN, W. 2015. Determining Antioxidant Activities of Lactobacilli Cell-Free Supernatants by Cellular Antioxidant Assay: A Comparison with Traditional Methods. *PLOS ONE,* 10**,** e0119058.
